# Supplementary material for: Epistatic models predict mutable sites in SARS-CoV-2 proteins and epitopes
Source: Proc Natl Acad Sci U S A. 2022 Jan 12;119(4):e2113118119. doi: 10.1073/pnas.2113118119 (PMC8795541; doi:10.1073/pnas.2113118119)
Supplement: Supplementary File [file pnas.2113118119.sapp.pdf]

Supplementary Information

Epistatic models predict mutable sites in SARS-CoV-2 proteins and epitopes

Juan Rodriguez-Rivas[1], Giancarlo Croce[2,3], Maureen Muscat[1], Martin Weigt*[1]

[1] Sorbonne Université, CNRS, Institut de Biologie Paris Seine, Computational and Quantitative Biology – LCQB, Paris, France.

[2] Department of Oncology, Ludwig Institute for Cancer Research Lausanne, University of Lausanne, Switzerland

[3] Swiss Institute of Bioinformatics (SIB), Lausanne, Switzerland

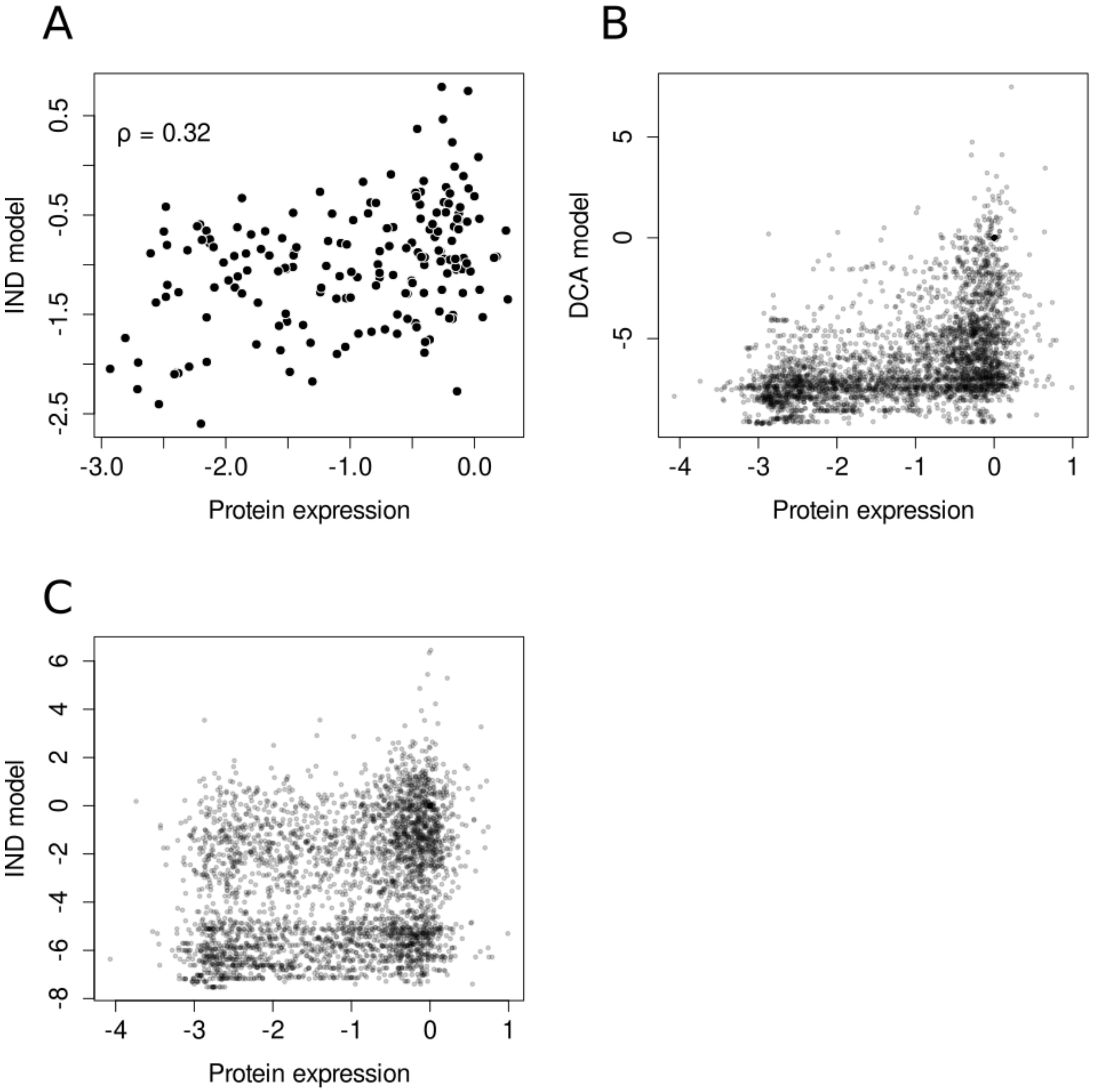

Fig. S1 A) Experimental protein expression for the 178 positions of the RBD as a function of the predicted effect by the IND model. The effects of 3355 single mutations in the 178 positions of the RBD measured by the experimental protein expression in the x-axis and predicted by the DCA model (B) and the IND model (C) in the y-axis.

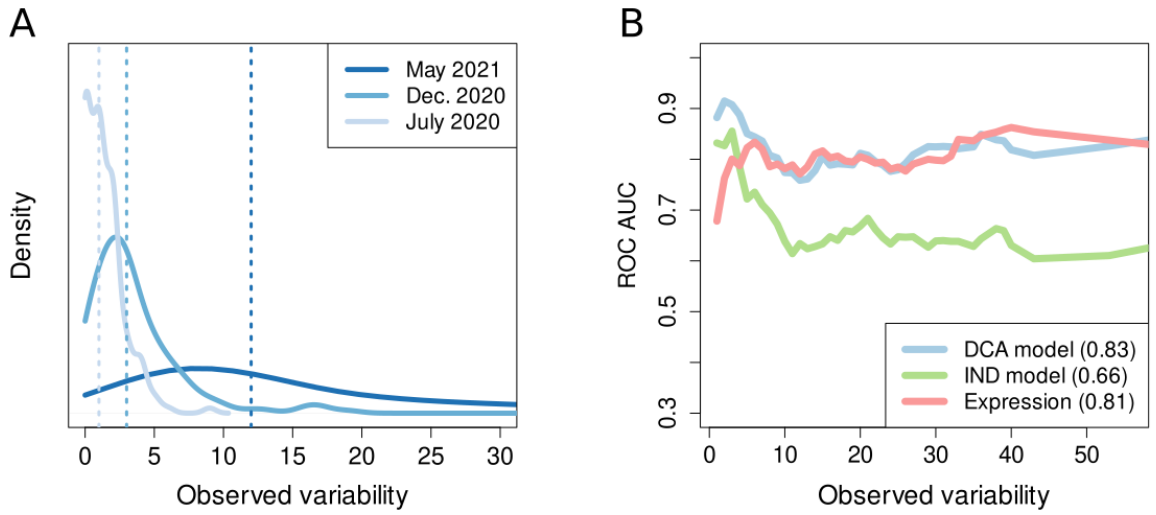

Fig. S2. A) Distributions of observed variability using the genomes available at July 2020, December 2020 and May 2021. The vertical dashed lines represent the median of each distribution and is used as a cutoff to distinguish between low- and high-variability positions. B) AUC for ROC curves using cutoffs of variability in the interval [1,56]. The mean AUC is shown in the legend.

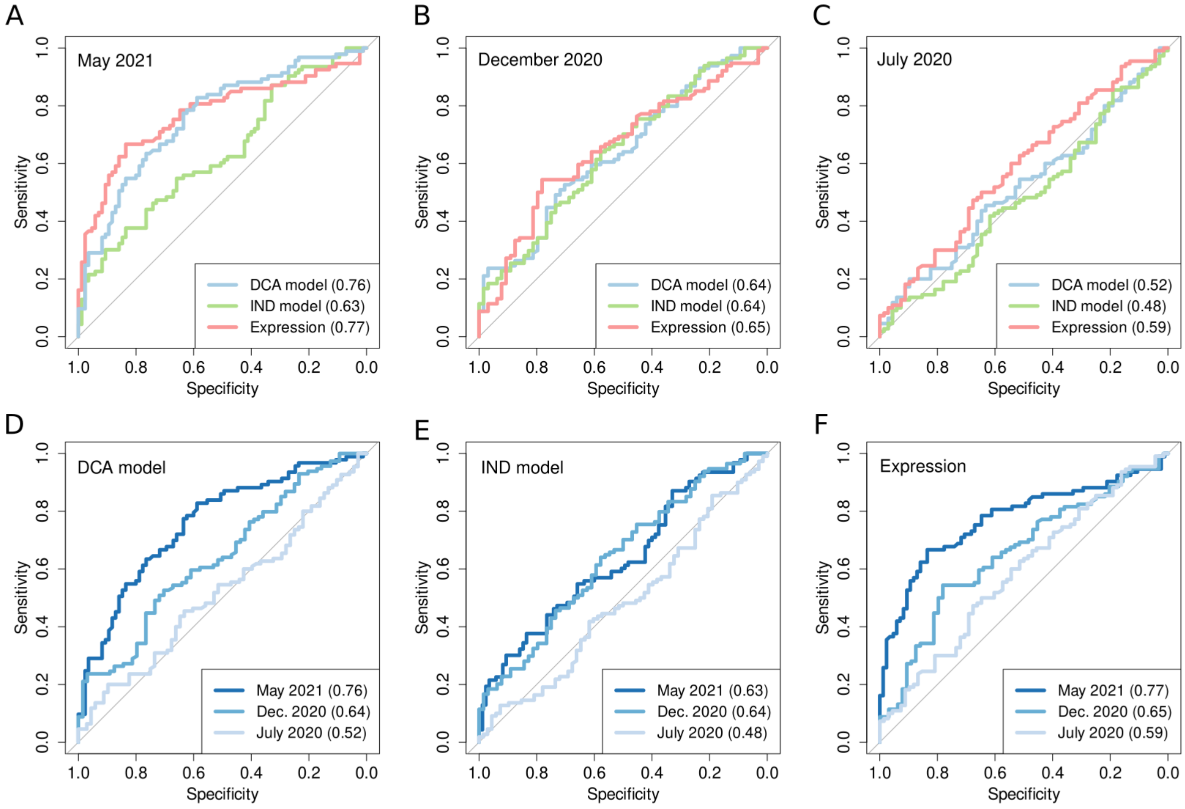

Fig S3. ROC curves for positions with low versus high observed variability for the 3 predictors with observed variability derived from all the SARS-CoV-2 genomes available at May 2021 (A), December 2020 (B), and July 2020 (C). ROC curves for positions with low versus high observed variability, where the observed variability is quantified with the SARS-CoV-2 genomes available at July 2020, December 2020, and May 2021 for the prediction coming from the DCA model (D), IND model (E), and protein expression (F).

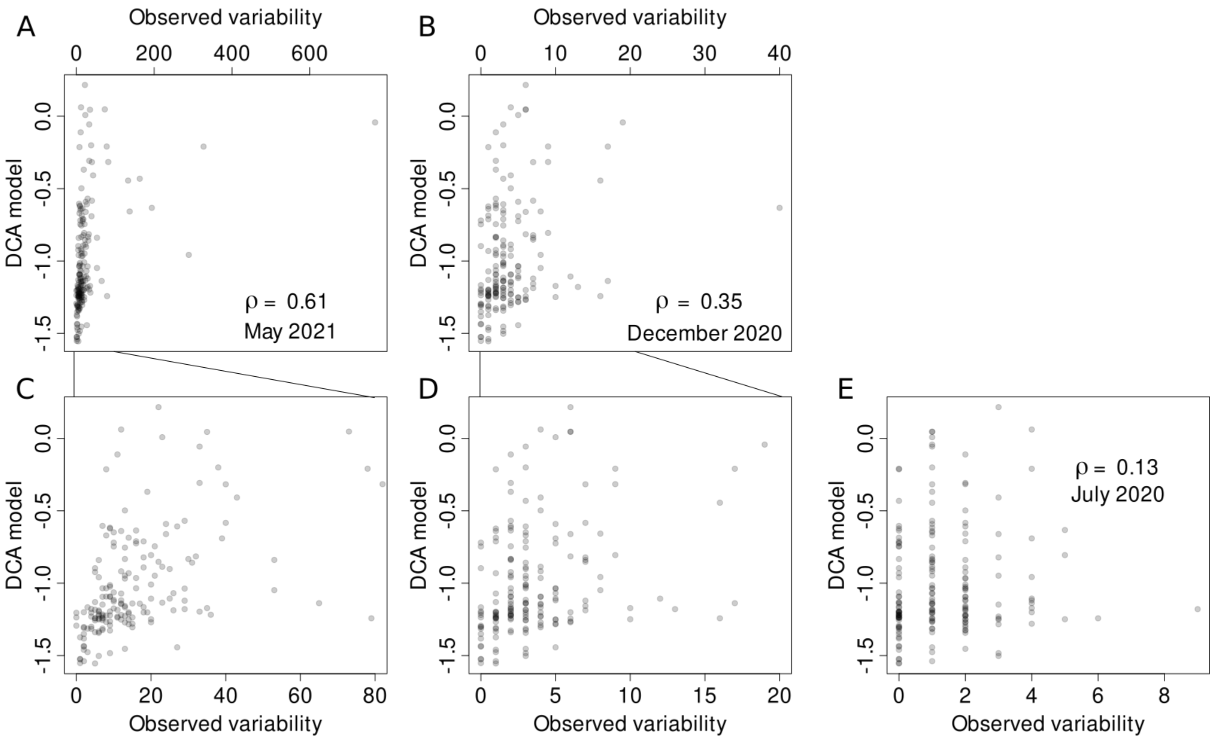

Fig. S4. Observed variability (estimated with GISAID data till May 2021, panels A and C; December 2020, B and D; July 2020, E) compared to the DCA predictions. The whole range of observed variabilities is shown in panels A, B, and E. In panels C and D, a more restricted interval of observed variabilities is shown to improve the visibility.

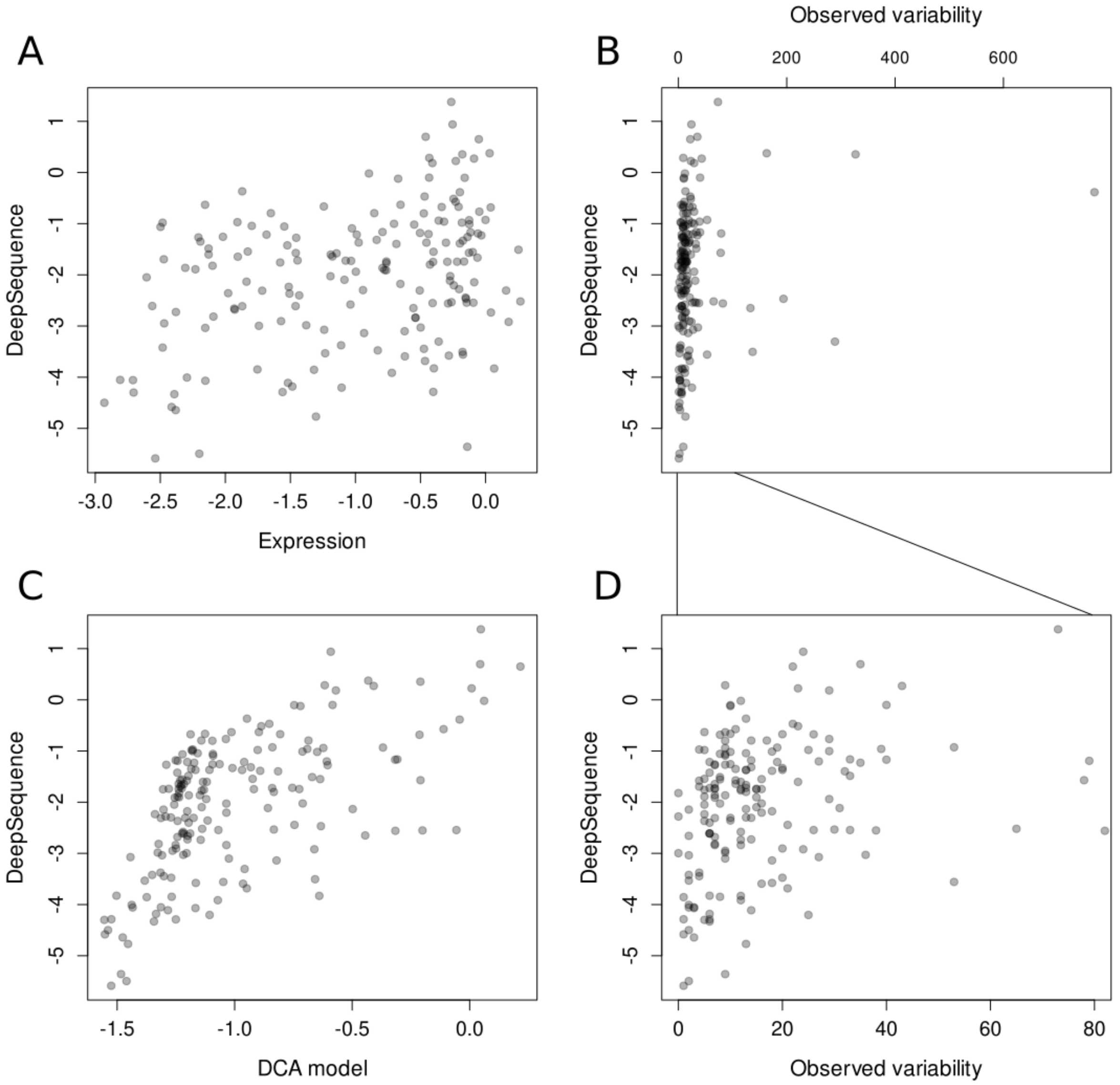

Fig. S5. A) Scatter plot between protein expression and DeepSequence scores. Comparison between observed variability and DeepSequence scores for the whole range of observed variability in May 2021 (B) and in the interval of [0,80] (D). C) DeepSequence scores as a function of the DCA model scores.

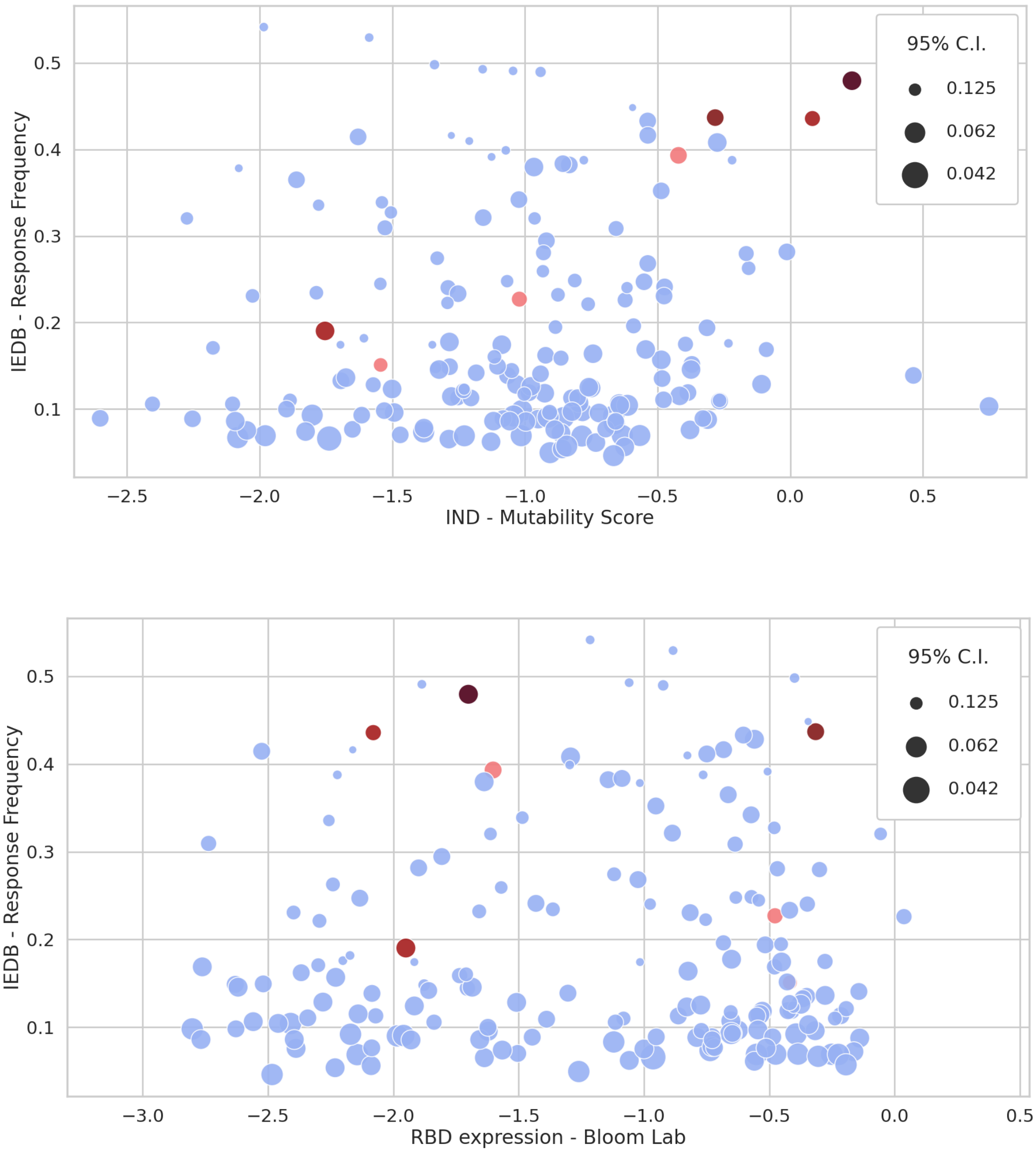

Fig. S6. The IEDB-Response Frequency as a function of the IND mutability score (upper panel) or protein expression (lower panel) for each position of the RBD domain. The enrichment of VOC/VOI mutations becomes less pronounced as compared to the DCA score.

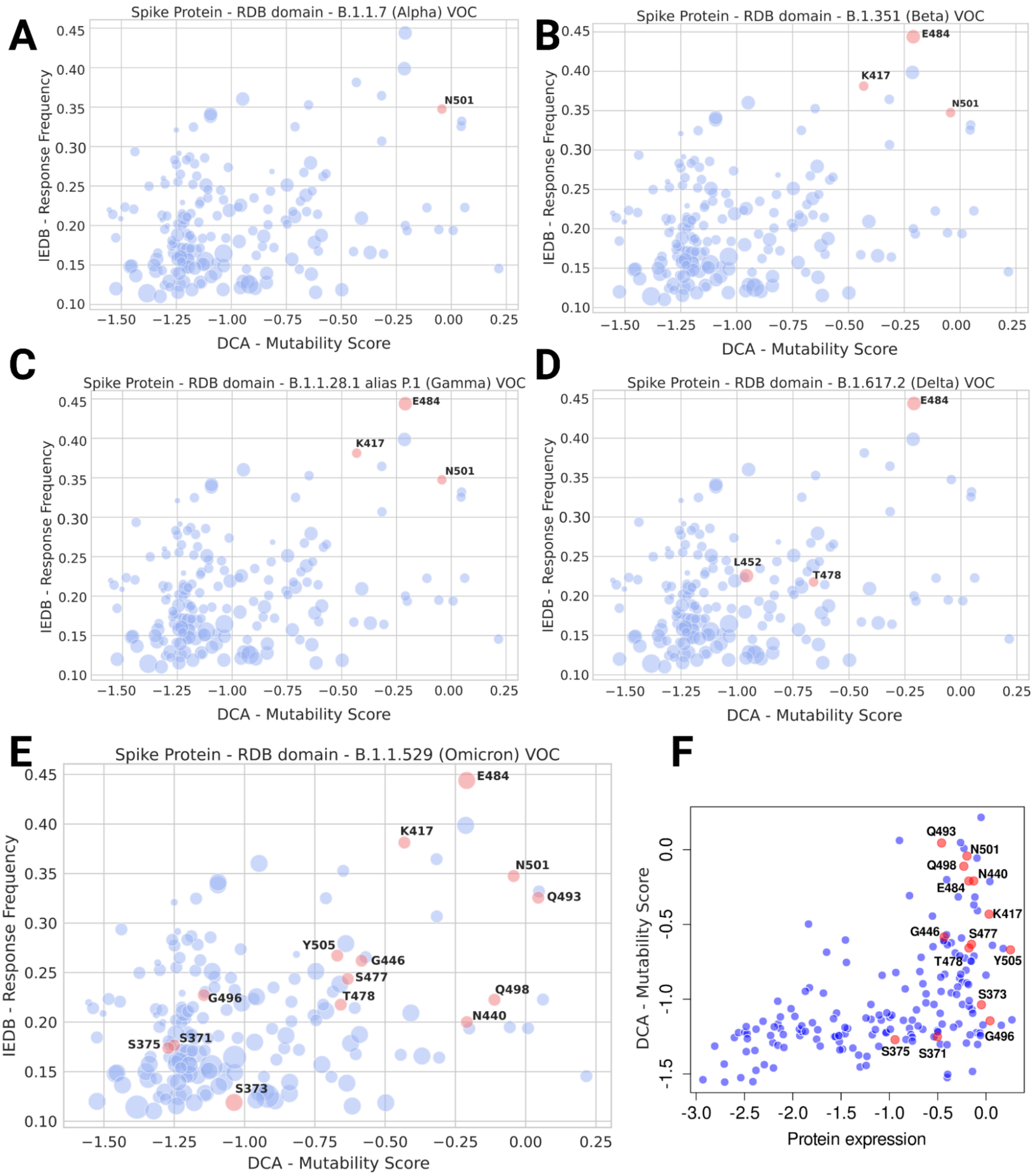

Fig. S7 - The IEDB-Response Frequency versus the DCA mutability score with updated IEDB response frequencies (data download 22 Nov 2021). We highlight in red the positions that are mutated in the 5 current VOCs, as of Dec 2021, indicated in https://cov-lineages.org/index.html: B.1.1.7 Alpha (Panel A), B.1.351 Beta (Panel B), P.1 Gamma (Panel C), B.1.617.2 Delta (Panel D), B.1.1.529 Omicron (Panel E) . We observe a pronounced enrichment for the Omicron variant in the upper right corner, i.e. positions that are likely to mutate (high DCA score), and whose mutations may cause immune escape (high IEDB - RF). Interestingly, the model predicts the positions S371, S373, S375, G496 - mutated in the Omicron variant (Panel E) - to be deleterious, even if they are neutral in the expression experiments (Panel F). As discussed in the main text, this is likely to be due to (a) limited datasets of functional sequences or (b) mutations without effect on expression that may still be deleterious for overall protein fitness. Currently available data are not able to discriminate between these two possibilities.

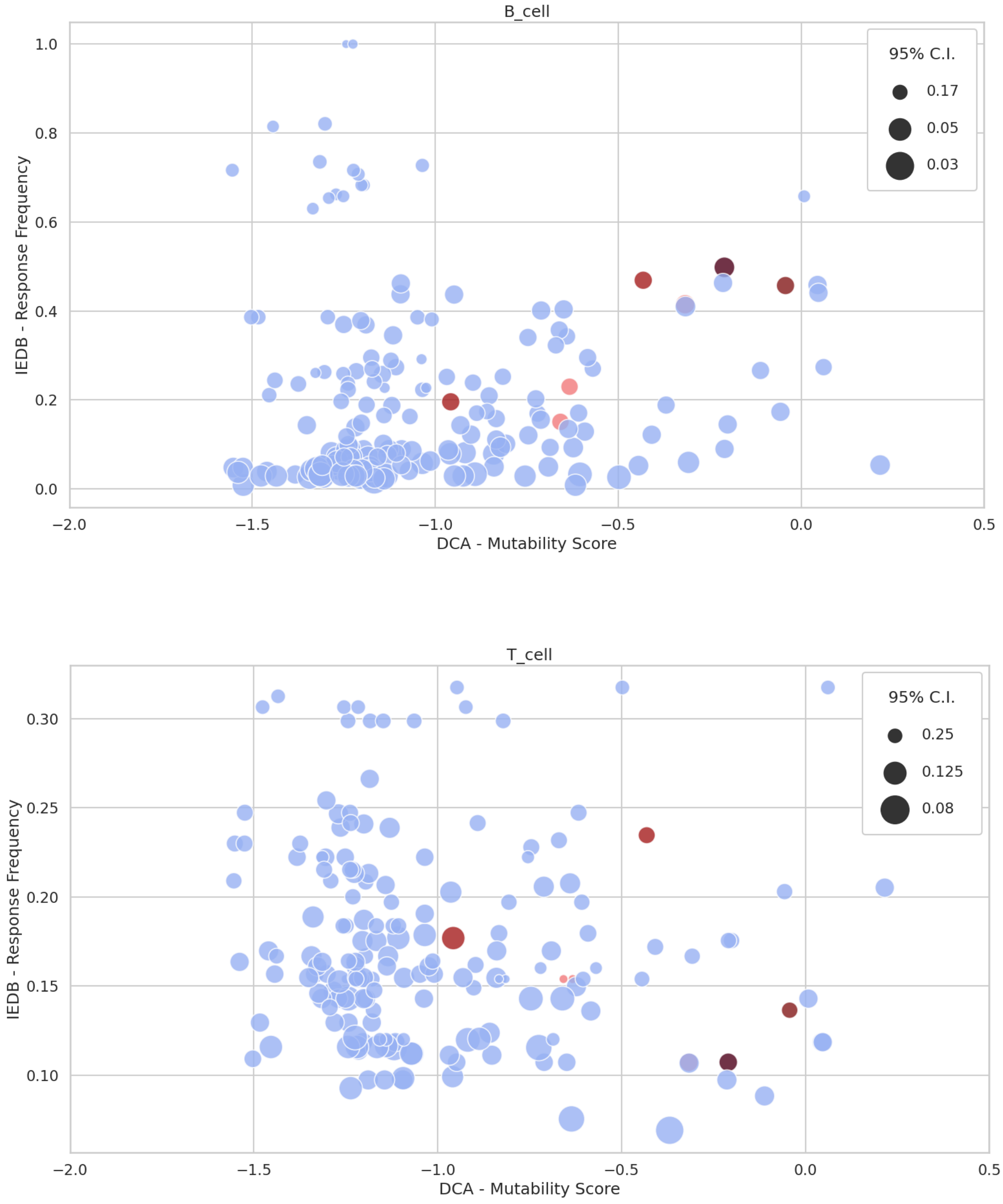

Fig S8. The IEDB-response frequency considering only B (upper panel) and T (lower panel) cell epitopes, and the DCA mutability score for each position of the RBD domain.

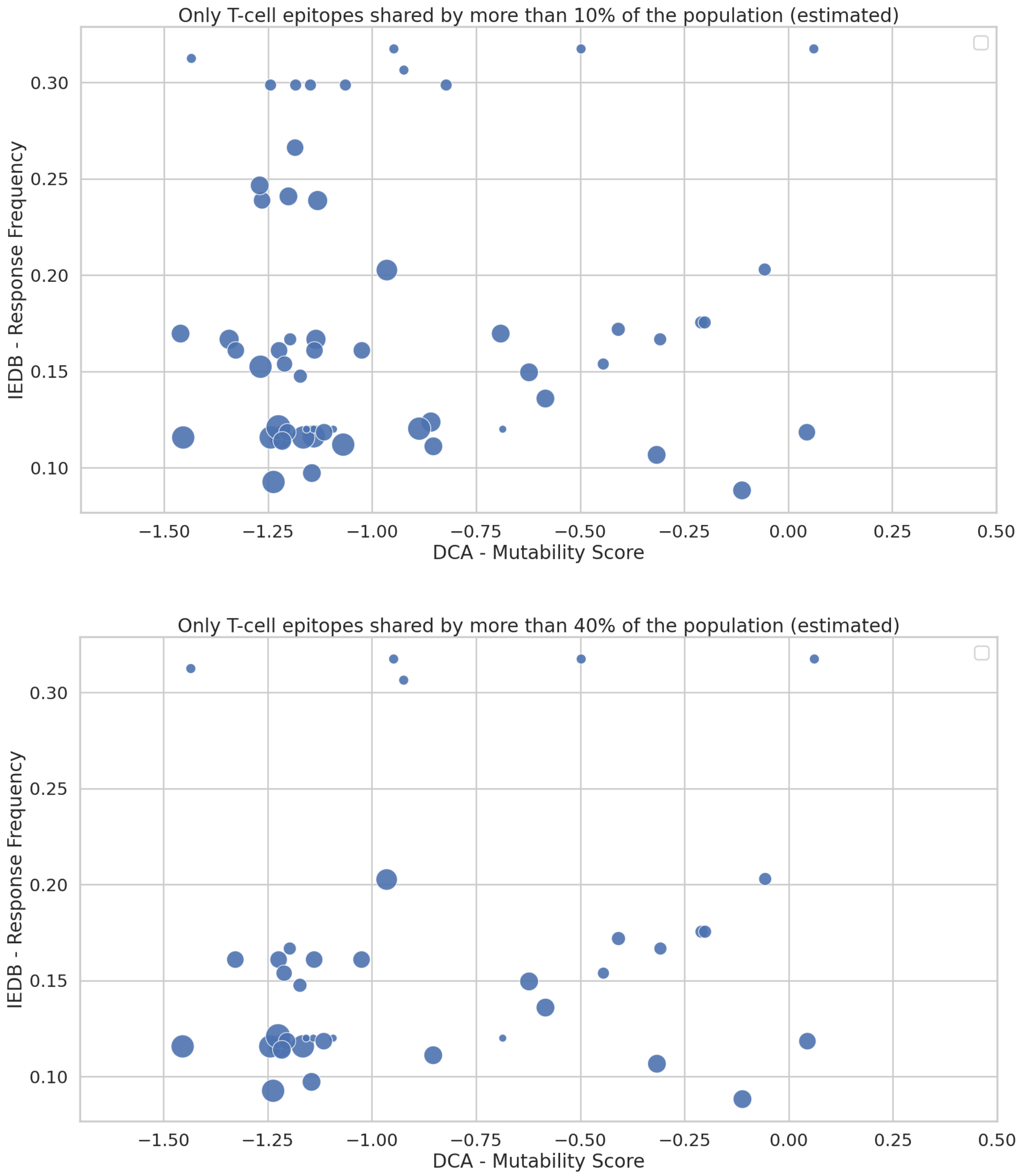

Fig S9. T cell immunoprevalent epitopes, i.e. predicted to be shared by at least the 10%(upper panel) and 40%(lower panel) of the world population. No clear correlation patterns between DCA and the IEDB emerge with the data available to date. Interestingly, no positions mutated in VOIs/VOCs were identified in T cell immunoprevalent epitopes.

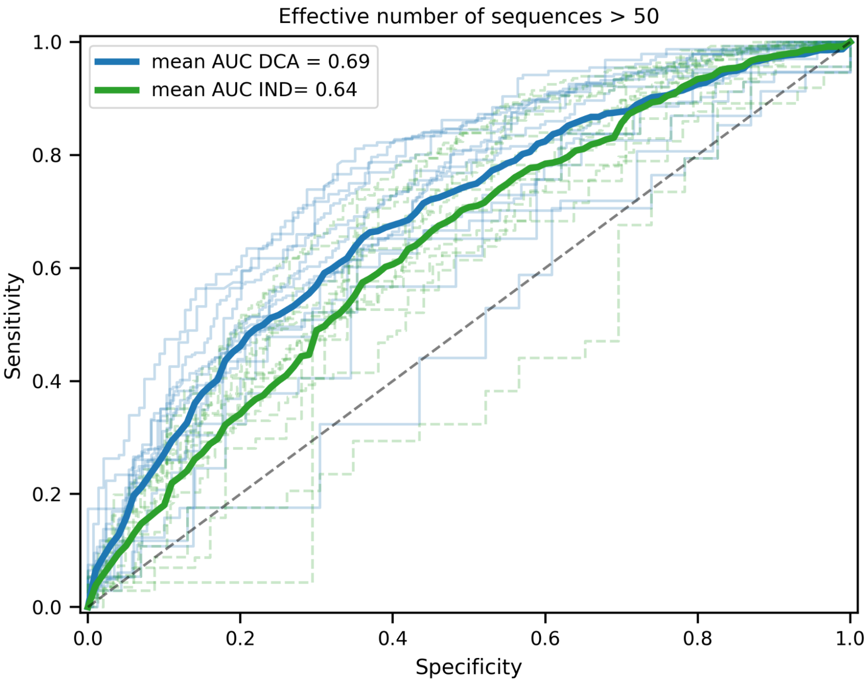

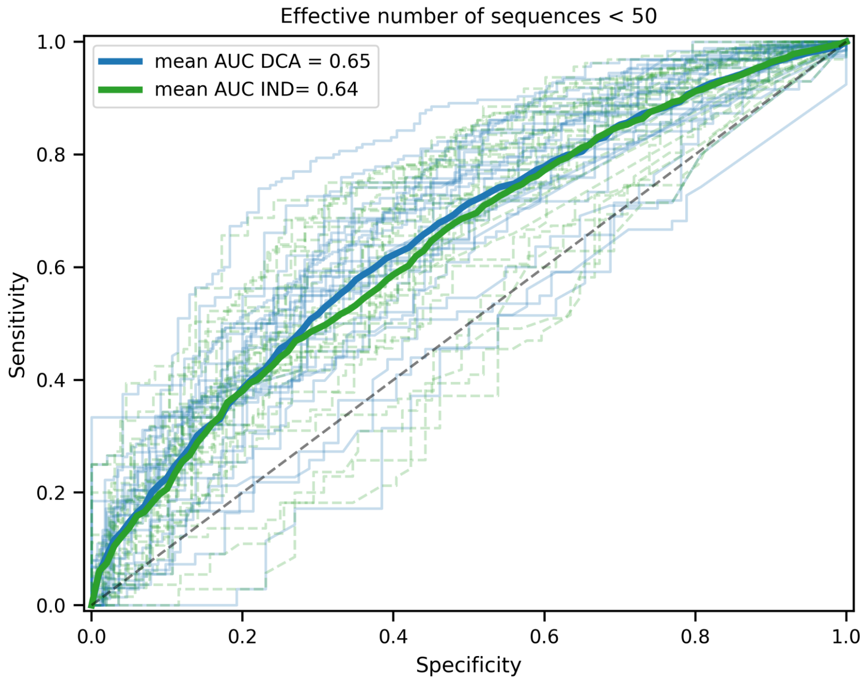

Fig S10. ROC curve for the DCA (blue) and IND (green) models for all 39 PFAM domains of the SARS-CoV-2 proteome, dividing between (upper panel) domains with more than 50 effective sequences (17 domains, 3491 positions) and (lower panel) less than 50 effective sequences (26 domains, 4546 positions). In bold, the mean ROC curves.

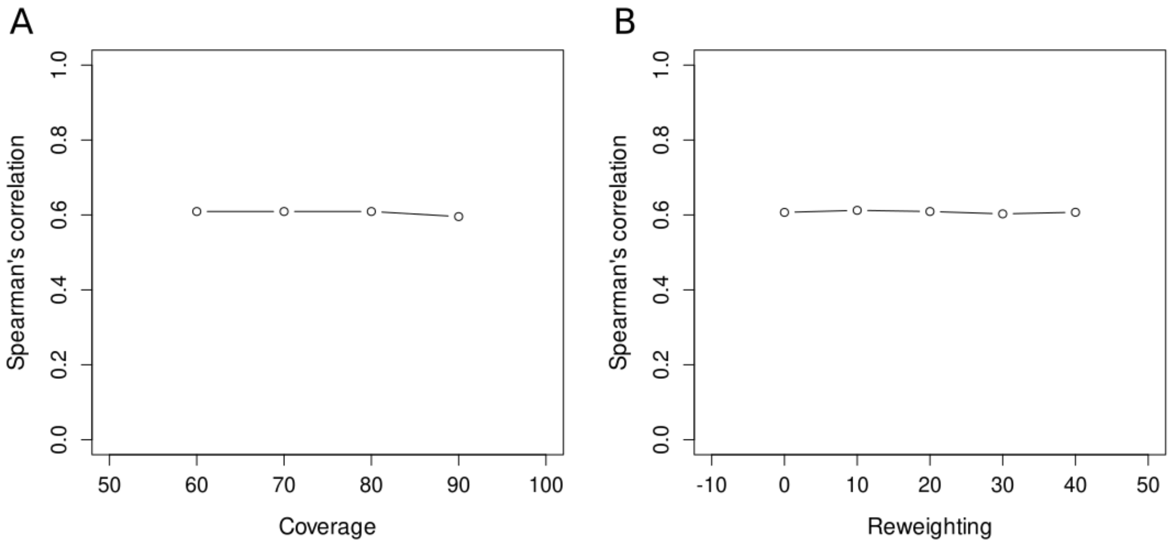

Fig S11. Spearman's correlation between the DCA model and the observed variability using different thresholds of coverage (filtering out sequences that do not cover that fraction of the reference sequence) (A) or the reweighting parameter (B) for model training.

Table S1. List of Pfam protein domains in the SARS-CoV-2 proteome, and the number of effective sequences and positions in the corresponding MSA

Protein/ORF	Pfam identifier	Pfam accession	N. eff. seq.	N. positions
Envelope	CoV_E	PF02723.15	53	66
Membrane	CoV_M	PF01635.19	40	201
Nucleocapsid	CoV_nucleocap	PF00937.19	48	341
ORF1a	bCoV_NAR	PF16251.6	19	98
ORF1a	bCoV_NSP1	PF11501.9	12	135
ORF1a	bCoV_NSP3_N	PF12379.9	9	171
ORF1a	bCoV_SUD_C	PF12124.9	2	64
ORF1a	bCoV_SUD_M	PF11633.9	10	143
ORF1a	CoV_NSP10	PF09401.11	25	123
ORF1a	CoV_NSP2_C	PF19212.1	22	167
ORF1a	CoV_NSP2_N	PF19211.1	25	241
ORF1a	CoV_NSP3_C	PF19218.1	58	488
ORF1a	CoV_NSP4_C	PF16348.6	33	96
ORF1a	CoV_NSP4_N	PF19217.1	58	354
ORF1a	CoV_NSP6	PF19213.1	71	262
ORF1a	CoV_NSP7	PF08716.11	31	83
ORF1a	CoV_NSP8	PF08717.11	30	197
ORF1a	CoV_NSP9	PF08710.11	33	113
ORF1a	CoV_peptidase	PF08715.11	103	319
ORF1a	Macro	PF01661.22	10075	107
ORF1a	Peptidase_C30	PF05409.14	41	291
ORF1b	CoV_Methyltr_1	PF06471.13	27	522
ORF1b	CoV_Methyltr_2	PF06460.13	31	296
ORF1b	CoV_NSP15_C	PF19215.1	42	153
ORF1b	CoV_NSP15_M	PF19216.1	40	97
ORF1b	CoV_NSP15_N	PF19219.1	38	61
ORF1b	CoV_RPol_N	PF06478.14	31	352
ORF1b	RdRP_1	PF00680.21	61	489
ORF1b	Viral_helicase1	PF01443.19	81660	225
ORF3a	bCoV_viroporin	PF11289.9	3	274
ORF6	bCoV_NS6	PF12133.9	4	61
ORF7a	bCoV_NS7A	PF08779.11	8	106
ORF7b	bCoV_NS7B	PF11395.9	3	42
ORF8	bCoV_NS8	PF12093.9	4	118
Spike	bCoV_S1_N	PF16451.6	103	305

Spike	bCoV_S1_RBD	PF09408.11	83	178
Spike	CoV_S1_C	PF19209.1	6231	57
Spike	CoV_S2_C	PF19214.1	50	40
Spike	CoV_S2	PF01601.17	79	522

Table S2. Pfam domains and the number of effective sequences in the MSAs obtained starting with full-length protein sequence or the domain sequence. In bold, the domains where there is a substantial difference.

Protein/ORF	Pfam identifier	N. eff. seqs. full-length	N. eff. seqs. domain
Envelope	CoV_E	53	49
Membrane	CoV_M	40	37
Nucleocapsid	CoV_nucleocap	48	47
ORF3a	bCoV_viroporin	3	3
ORF6	bCoV_NS6	4	4
ORF7a	bCoV_NS7A	8	8
ORF7b	bCoV_NS7B	3	3
ORF8	bCoV_NS8	4	4
**Spike**	**bCoV_S1_N**	**103**	**48**
**Spike**	**bCoV_S1_RBD**	**83**	**26**
**Spike**	**CoV_S1_C**	**123**	**6231**
**Spike**	**CoV_S2_C**	**50**	**7**
Spike	CoV_S2	78	79

Table S3. Strongest inter-domain epistatic couplings for pairs of domains with a maximum (out of all the possible inter-domain epistatic couplings between the pair of domains) coupling higher than 0.5.

First protein/ORF	First domain	Second protein/ORF	Second domain	Max. coupling
ORF1a	CoV_NSP2_N	ORF3a	bCoV_viroporin	0.85
ORF3a	bCoV_viroporin	ORF8	bCoV_NS8	0.63
ORF1a	CoV_RPol_N	Spike	bCoV_viroporin	0.57
ORF1b	CoV_NSP2_N	ORF3a	bCoV_S1_RBD	0.56

SI text

Sequence data

Sequence data in FASTA format were downloaded from the following databases: GISAID (release 16 May 2021), Uniref90 (ref, release December 2020), ViPR (downloaded in September 2020), NCBI viral genomes (downloaded in September 2020) and MERS coronavirus database (downloaded in September 2020). The amino acid sequence of isolate Wuhan-Hu-1 was used as the reference proteome (genbank identifier: MN908947). Protein domains were detected using the HMMER suite (ref, version 3.1b2) and the HMM profiles from Pfam. After running the command *hmmsearch* from the HMMER suite (ref, version 3.1b2) on the reference proteome using the HMM profiles of SARS-CoV-2 provided by Pfam, the domain amino acid sequence of the full-length protein were trimmed accordingly to the *hmmsearch* output to obtain a reference sequence for each domain. We kept all non-overlapping pfam domains with a domain e-value lower than 10^{-5}.

A global sequence database including distant species was built by combining Uniref90, ViPR, NCBI viral genomes and MERS coronavirus database. Starting with the domain sequences, we built MSAs by running *jackhmmer* with 5 iterations. For the proteins not belonging to the ORF1ab (which is too long to apply this procedure), we also built MSAs with *jackhmmer* with 5 iterations starting with the full-length reference protein sequences instead of domain sequences. The resulting full-length protein MSAs were decomposed and trimmed to domain alignments by keeping the corresponding columns. When two MSAs from the global database were obtained (one coming from the full-length sequence and another coming from the domain sequence), the one with the highest number of sequences non-redundant at 80% was kept for further analysis for each Pfam domain. Although both strategies usually recover a similar number of sequences, there exists a substantial difference for most domains in the Spike protein (Table S2), allowing us to increase the available sequence data in their MSAs. As quality controls, all sequences including non-standard amino acids were removed as well as repeated sequences or sequences covering less than 80% of the reference domain sequence. To avoid a bias toward the reference sequence, all sequences closer than 90% sequence identity to the Wuhan-Hu-1 reference were filtered out.

For the GISAID database, an MSA for each domain sequence was built using the command *jackhmmer* from the HMMER suite with only 1 iteration as the GISAID sequences are very similar to those in the reference proteome. We filtered sequences including non-standard amino acids, coverage lower than 80%, and those belonging to a non-human host. Only non-identical sequences were considered to avoid the strong sequencing bias due to the highly diverse number of genomes sequenced in different countries. The variability of each position was estimated by counting the number of sequences that have a different amino acid in the corresponding position compared to the reference.

Co-alignments of domains

Starting with the 39 raw alignments of domains constructed using both the alignments of distant and close sequences (*Materials and methods*), we build 741 co-alignments (all possible combinations of two domains) by joining the sequences coming from the same genome (thanks to the genome accession number). Co-alignments with fewer than 50 effective sequences were discarded to increase the reliability of the predictions. Note that the number of effective sequences is higher in this analysis compared to the mutability predictions because of the large number of close sequences. From the remaining 601 co-alignments, we computed the models as in case of single domains (see *Material and methods*) and obtained the APC scores from the DCA model between each pair of inter-domains positions. The pairs of domains with at least one APC score higher than 0.3 are linked in Fig. 4D. The 4 predictions with the highest APC scores can be found in Table S3.
